# How Reliably Do Large Language Models Reproduce Vital Pulp Therapy Guidelines? A Mixed-Effects Evaluation of Guideline-Concordance and Error Directionality

**DOI:** 10.3390/healthcare14121605

**Published:** 2026-06-07

**Authors:** Sine Güngör Us, Arzu Şahin Mantı, Arzu Kaya Mumcu

**Affiliations:** 1 Department of Endodontics, Faculty of Dentistry, Gazi University, 06490 Ankara, Turkey; 2 Private Practice, 06490 Ankara, Turkey; dtarzusahin@hotmail.com; 3 Department of Endodontics, Faculty of Dentistry, Kütahya Sağlık Bilimleri University, 43100 Kütahya, Turkey; arzu.kayamumcu@ksbu.edu.tr

**Keywords:** large language models, guideline-concordance, vital pulp therapy, prompt engineering, error directionality, mixed-effects logistic regression

## Abstract

**Highlights:**

**What are the main findings?**

- High guideline-concordance accuracy does not guarantee uniform clinical reliability; LLMs showed distinct false-positive and false-negative tendencies that varied by model and prompt condition.
- Short-term response stability was observed across all models, but professional-role prompting shifted error profiles in model-specific and sometimes adverse directions.

**What are the implications of the main findings?**

- Evaluating LLMs in protocol-sensitive clinical domains requires multidimensional assessment beyond average accuracy, including error directionality, prompt responsiveness, and item-level variability.
- Clinicians consulting LLMs for guideline-based decisions should consider model-specific error tendencies rather than relying solely on reported accuracy benchmarks.

**Abstract:**

**Background**: Large language models (LLMs) are increasingly consulted for clinical guidance, yet their reliability in protocol-sensitive domains remains insufficiently characterized. This study evaluated the ability of widely accessible LLMs to reproduce guideline-defined decision thresholds in vital pulp therapy (VPT), with emphasis on guideline-concordance accuracy, professional-role prompting, short-term response stability, and decision-level error directionality. **Methods**: Twenty-six binary yes/no questions were derived from an internationally recognized evidence-based guideline for VPT. Four LLMs—GPT-5, GPT-4o, DeepSeek-V3, and Gemini 2.5 Flash—were queried under non-prompted and professional-role-prompted conditions by two independent operators across three daily sessions over three consecutive days. Descriptive analyses were complemented by mixed-effects logistic regression in R to account for repeated responses clustered within guideline-derived questions. **Results**: Overall guideline-concordance accuracy was high across models. Gemini showed the highest observed accuracy under non-prompted conditions; DeepSeek showed the highest under prompted conditions. In the mixed-effects model, Gemini demonstrated significantly higher odds of guideline-concordant responses than GPT-5 under non-prompted conditions, whereas DeepSeek outperformed GPT-5 and GPT-4o under prompted conditions. The model × prompt interaction showed a trend toward significance but did not reach the conventional threshold. Day and within-day time point were not significantly associated with accuracy, supporting short-term response stability. Error-direction analysis revealed model-specific patterns: Gemini showed consistently low false-positive rates but increased false-negative responses under prompted conditions; DeepSeek showed reduced false-positive and no false-negative responses under prompted conditions. **Conclusions**: Average accuracy alone is insufficient to characterize the reliability of LLM-generated clinical guidance. Evaluation in protocol-sensitive domains should incorporate guideline-concordance, prompt responsiveness, short-term stability, and decision-level error directionality.

## 1. Introduction

Artificial intelligence (AI), particularly large language models (LLMs), has rapidly expanded into clinical information retrieval and decision-support workflows. These systems generate context-aware responses to natural-language input to produce guidance that can approximate expert reasoning [1,2,3]. As LLM-based chatbots such as ChatGPT, DeepSeek, and Gemini become widely accessible, they are increasingly consulted for diagnostic and therapeutic recommendations across healthcare disciplines [2].

AI-based applications have been increasingly integrated into various areas of dentistry—including restorative dentistry, orthodontics, periodontology, and oral and maxillofacial surgery—to support guideline-based decision-threshold reproduction, guide treatment planning, and assist in prognosis assessment [4,5,6,7]. In endodontics, AI tools have demonstrated strong performance in detecting root fractures, evaluating periapical pathologies, determining working length, and analyzing root morphology [8,9]. LLMs now provide rapid access to endodontic knowledge to support clinical decision-making, and offer personalized learning opportunities for students [10,11].

Despite this growing use, their performance in real clinical settings remains uncertain. In response to identical clinical queries, LLMs exhibit variability and limited reproducibility across repeated trials [12], raising concerns regarding reliability, error patterns, and inconsistent guidance [13]. Among endodontic procedures, vital pulp therapy (VPT) is increasingly prominent due to an increasing emphasis on conservative, biologically oriented treatment approaches. While common, VPT requires strict adherence to diagnostic and procedural criteria, making it particularly sensitive to misinformation and reasoning errors. Because treatment decisions depend on nuanced parameters—such as pulpal inflammation status, hemostasis quality, exposure etiology, biomaterial selection, and restoration protocols—LLM-related errors may negatively affect clinical decisions [14]. Importantly, such errors may occur in different directions. A false-positive response—endorsing a guideline-defined clinical rule when it is not applicable—may promote inappropriately conservative approaches, whereas a false-negative response may result in unnecessary treatment escalation or abandonment of a potentially successful conservative option. Because treatment decisions in VPT depend on nuanced diagnostic and procedural parameters, directionality of errors may have clinically relevant implications for decision support.

Assessments of LLM performance in endodontics have primarily focused on overall guideline-concordance accuracy [15]. Some investigations have explored prompt-related effects or response consistency in isolation; however, these dimensions are often evaluated separately and within limited experimental settings [12,16,17]. Consequently, aspects such as short-term response variability, the directional tendencies of errors, and cross-session stability remain insufficiently characterized. This limits our understanding of whether LLMs can consistently support clinical decision-making across different contexts. The novelty of this study lies not in the use of newly developed LLM architectures, but in the multidimensional evaluation framework applied to a protocol-sensitive clinical domain. Unlike generic chatbot accuracy comparisons, this study evaluates whether widely accessible LLMs can reproducibly recover guideline-defined VPT decision thresholds across repeated sessions, operators, and prompt conditions. By combining professional-role prompting, short-term stability assessment, decision-level false-positive and false-negative error-directionality profiling, and mixed-effects modelling that accounts for item-level clustering, this study provides a structured framework for evaluating the reliability of LLM-generated guideline consultation beyond average accuracy.

Building on this rationale, the primary aim of the present study was to evaluate the reliability of widely accessible LLMs in reproducing guideline-defined VPT decision thresholds, operationalized as a composite of guideline-concordance accuracy, short-term response stability, professional-role prompt responsiveness, and decision-level error directionality. Secondary aims included between-model comparison of these dimensions and characterization of model-specific error profiles under non-prompted and professional-role-prompted conditions. A multi-session and multi-operator framework was adopted to enable a granular assessment beyond overall accuracy. The null hypothesis was that there would be no significant differences between the evaluated LLMs in guideline-concordance accuracy, prompt responsiveness, short-term stability, or decision-level error directionality.

## 2. Materials and Methods

### 2.1. Ethical Considerations and Study Design

This comparative cross-sectional study evaluated the accuracy and reliability of responses generated by LLMs to standardized questions regarding VPT. The study involved no human participants or identifiable data; therefore, ethical approval was not required. Procedures were conducted in accordance with TRIPOD-LLM guidelines and general research integrity principles.

### 2.2. Chatbot Selection and Experimental Conditions

Four widely used, up-to-date LLMs were evaluated in their premium and most recent versions:

- ChatGPT-5 (OpenAI, San Francisco, CA, USA) [18]
- ChatGPT-4o (OpenAI, San Francisco, CA, USA) [19]
- DeepSeek V3 (DeepSeek AI, Hangzhou, China) [20]
- Gemini Advanced 2.5 Flash (Google, Mountain View, CA, USA) [21]

These models represent the most widely used, currently accessible, state-of-the-art LLM chatbots.

All models were accessed through publicly available web interfaces using premium subscription versions, ensuring availability of the most current model configurations at the time of testing.

All model interactions were conducted between October 31 and 2 November 2025, using the publicly available web interfaces. This defined testing period ensured that model performance reflected a fixed system state during data collection.

Two testing conditions were applied:

1. Non-prompted condition
2. Professional-role-prompted condition with the standardized instruction: “You are an experienced endodontist.”

All models were operated using default system parameters without manual adjustment of temperature, top-p, or token limits. These parameters were not externally configurable in the web interface and were kept constant across all sessions.

### 2.3. Question Development and Content

A total of 26 binary (yes/no) decision statements were developed based on an internationally recognized evidence-based clinical guideline for the management of deep caries and exposed pulp [22], following a structured three-stage process. First, two endodontists (S.G.U. and A.Ş.M.) independently reviewed the source guideline section by section and extracted candidate decision points covering diagnostic criteria, indications, hemostasis parameters, disinfection protocols, material selection, restoration principles, and follow-up recommendations. Each candidate decision point was reformulated as a binary yes/no statement representing a single, guideline-defined rule. Second, the two item sets were merged; discrepancies in wording, scope, or content were resolved through item-by-item consensus discussion to ensure that each retained statement mapped directly to an explicit guideline recommendation rather than to indirect inference. Third, the consolidated item set was reviewed by a senior endodontist (>5 years of clinical experience), who evaluated content validity against the source guideline, edited individual item wording where needed for precision and clarity, and provided recommendations that were incorporated into the final question set. Gold-standard answers were defined strictly according to the published guideline criteria, with no inferential extension beyond explicit recommendations. All questions were formulated in English, reflecting the language of the source guideline. The complete list of all decision statements and corresponding gold-standard answers is provided in Supplementary Table S1.

### 2.4. Data Collection Procedure

Two independent operators administered each question at three fixed daily time points (morning, afternoon, evening) under both testing conditions, producing 12 responses per question per model per day. Data collection continued over three consecutive days, yielding 36 responses per question per model. The three-consecutive-day window was selected as a pragmatic design choice intended to generate sufficient repeated observations for characterization of short-term response stability while keeping data collection within a fixed system state. This window is broadly consistent with comparable short-term LLM evaluation designs in endodontics [12,23].

To preserve operator independence during data collection, each operator used a separate device and a separate user account on each LLM platform, executed all queries from an identical pre-prepared question list to eliminate operator-driven variation in wording, and recorded responses independently into a structured spreadsheet template without exchanging any model outputs during the three-day testing period. Although both operators had access to the source guideline for the subsequent response-coding step, they administered queries and recorded outputs independently and did not communicate about specific model responses during the data collection phase.

To prevent contextual carryover within or between testing days, a new chat session was initiated for every individual query, such that no question was administered within an active session containing prior responses. Previous conversations were neither referenced nor continued, and each query was submitted as a fresh, independent input. The exact wording and punctuation of each question were preserved across operators, time points, and prompt conditions.

LLM outputs were recorded as unstructured natural language text. Each response was subsequently classified into a binary outcome (correct/incorrect) by mapping the semantic content of the response to the corresponding gold-standard answer. Responses that included hedging language, conditional statements, or ambiguous phrasing without a clear directional stance were flagged and resolved through consensus between the two raters. Responses were coded as 1 (correct, matching gold standard) or 0 (incorrect) independently by two researchers. Incomplete, non-binary, or non-interpretable responses were identified according to predefined scoring rules. When a response could not be clearly categorized as yes or no, it was coded as non-concordant for the primary analysis unless the intended binary answer was explicitly inferable from the response. Such cases were documented separately for transparency.

### 2.5. Outcome Measures

The primary outcome was overall guideline-concordance accuracy, calculated as the proportion of chatbot responses that matched the established gold-standard answers. Several secondary outcomes were assessed, including the prompt effect, which measured the variance in accuracy between prompted and non-prompted conditions, and error directionality, assessing the distribution of false-positive versus false-negative responses. Furthermore, the reliability of the models was rigorously analyzed through within-day consistency—reflecting the agreement between morning, afternoon, and evening sessions—and short-term response stability, which tracked accuracy fluctuations across three consecutive days.

### 2.6. Statistical Analysis

Data were categorical and presented as frequencies and percentages. Since accuracy values did not meet normality assumptions, non-parametric tests were employed. The Kruskal–Wallis test was used to compare mean accuracy between models, followed by post hoc multiple comparisons where significant differences were detected. Within-model comparisons between non-prompted and prompted conditions were evaluated using the Wilcoxon signed-rank test. McNemar’s test assessed paired response shifts on a day-by-day basis. Inter-rater agreement between observers was assessed using Cohen’s kappa, and agreement levels were interpreted according to the classification proposed by Landis and Koch [24]. Short-term response stability was described using standard deviations (SD) of accuracy percentages across time points, as intraclass correlation coefficients (ICC) were deemed inappropriate for nominal data. Clinical risk profiles were characterized by calculating false-positive (FP) and false-negative (FN) rates for each model. Within-model prompt-related changes in FP and FN rates were explored using Fisher’s exact test.

In addition to descriptive and non-parametric analyses, a mixed-effects logistic regression model was fitted as a confirmatory analysis to account for the repeated-measures structure of the dataset. Since subsequent analyses were performed using Observer 1 data after confirmation of high inter-observer agreement, the mixed-effects model was also based on Observer 1 evaluations. Model type, prompt condition, the model × prompt interaction, day, and within-day time point were included as fixed effects. Question ID was included as a random intercept to account for clustering of repeated responses within the same guideline-derived item. Results were reported as odds ratios (ORs) with 95% confidence intervals. Bonferroni-adjusted pairwise marginal comparisons were used for between-model contrasts within each prompt condition. Model fit was evaluated by checking for overdispersion and singular fit.

An a priori sample size calculation specific to the interaction terms in the mixed-effects model was not performed, given the item-level clustering structure of the design and the absence of established effect-size benchmarks for guideline-concordance interaction effects in prior LLM evaluations in this domain. The total of 3744 responses, derived from 26 items repeated under multiple conditions, supported substantial power for between-model main effects, whereas precision for individual model × prompt interaction terms was lower, ref. [1] as reflected in the confidence intervals around those estimates.

Descriptive and non-parametric analyses were performed using IBM SPSS Statistics version 20.0 (IBM Corp., Chicago, IL, USA). Mixed-effects logistic regression and post-hoc pairwise comparisons were performed in R (version 4.6.0; R Foundation for Statistical Computing, Vienna, Austria, 2026) using the lme4 and emmeans packages. Statistical significance was set at *p* < 0.05.

## 3. Results

A total of 3744 individual responses were generated across all models (26 questions × 4 LLMs × 2 operators × 3 daily sessions × 3 consecutive days × 2 prompt conditions). All four language models completed the full question set without interruption under both prompted and non-prompted conditions.

Overall, inter-observer agreement was almost perfect across the majority of models and time points under both non-prompted and prompted conditions (κ > 0.81). A limited number of time-specific exceptions showed substantial agreement (Table 1).

Cohen’s kappa statistics reflect agreement between observers across all reported conditions. Agreement levels were interpreted according to the classification proposed by Landis and Koch [24].

Based on this high level of agreement, all subsequent analyses were conducted and reported using the evaluations of Observer 1.

### 3.1. Comparison of Guideline-Concordance Accuracy Between Models

#### 3.1.1. Non-Prompted Condition (NP)

Under non-prompted conditions, a significant difference in mean guideline-concordance accuracy was observed between models (*p* = 0.007). Performance metrics are presented in Table 2.

Among the evaluated models, Gemini demonstrated the highest mean guideline-concordance accuracy under non-prompted conditions. Post hoc pairwise comparisons indicated that Gemini performed significantly better than GPT-5 (*p* = 0.003), whereas no statistically significant differences were observed between the remaining model pairs.

#### 3.1.2. Prompted Condition (P)

Under prompted conditions, a significant difference in mean guideline-concordance accuracy was observed between models (*p* < 0.001; Table 3).

DeepSeek demonstrated the highest mean accuracy under prompted conditions, significantly higher than that of GPT-5 (*p* < 0.001) and GPT-4o (*p* = 0.002). No significant differences were observed between the remaining model pairs.

These values represent observed guideline-concordance accuracy. Model-adjusted comparisons accounting for repeated question-level clustering are presented in the mixed-effects logistic regression analysis (Table 4).

The model included chatbot model, prompt condition, model × prompt interaction, day, and within-day time point as fixed effects, with question ID included as a random intercept. GPT-5, non-prompted condition, Day 1, and morning session were used as reference categories. OR: odds ratio; CI: confidence interval.

**Figure 1:**
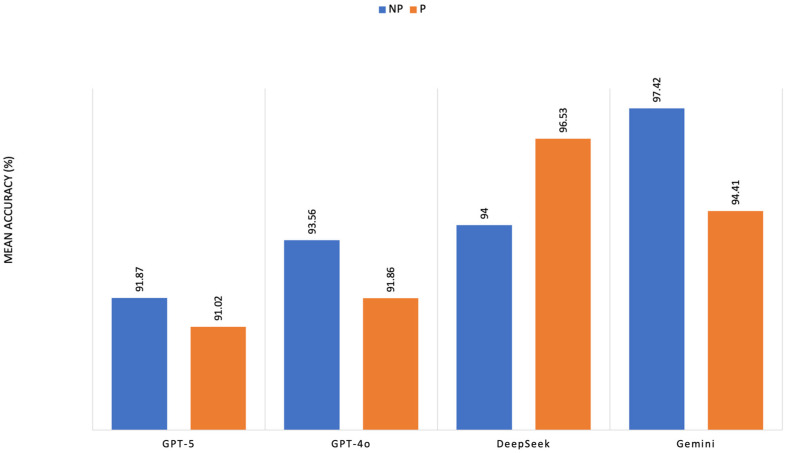
Comparison of mean guideline-concordance accuracy across chatbot models under non-prompted (NP) and prompted (P) conditions.

A comparative visualization of model performance across both conditions is provided in Figure 1.

### 3.2. Mixed-Effects Logistic Regression Analysis

To account for repeated responses clustered within the same guideline-derived questions, a mixed-effects logistic regression model was fitted using Observer 1 evaluations. The model included chatbot model, prompt condition, model × prompt interaction, day, and within-day time point as fixed effects, with question ID entered as a random intercept. The random intercept variance for question ID was 6.698, indicating substantial item-level variability across the guideline-derived questions.

In the main mixed-effects model, Gemini showed significantly higher odds of guideline-concordant responses than GPT-5 under non-prompted conditions (OR = 5.73, 95% CI: 1.90–17.30, *p* = 0.002; Table 4). GPT-4o and DeepSeek did not differ significantly from GPT-5 under non-prompted conditions. Prompt condition, day, and within-day time point were not significantly associated with guideline-concordant response accuracy.

The model × prompt interaction showed a trend toward significance but did not reach the conventional statistical threshold after accounting for question-level clustering (χ^2^ = 6.79, df = 3, *p* = 0.079). Within-model prompt comparisons did not demonstrate statistically significant prompt-related changes after adjustment. However, descriptive patterns suggested model-dependent prompt responsiveness: DeepSeek showed higher observed accuracy under prompted conditions, whereas Gemini showed lower observed accuracy under prompted conditions.

Bonferroni-adjusted pairwise comparisons were additionally performed within each prompt condition. Under non-prompted conditions, Gemini remained significantly superior to GPT-5 (Bonferroni-adjusted *p* = 0.012). Under prompted conditions, DeepSeek showed significantly higher odds of guideline-concordant responses than both GPT-5 (Bonferroni-adjusted *p* = 0.018) and GPT-4o (Bonferroni-adjusted *p* = 0.032; Table 4). No other between-model comparisons remained statistically significant after Bonferroni adjustment. The overall model × prompt interaction did not reach statistical significance, although individual interaction terms suggested model-dependent prompt responsiveness.

### 3.3. Error Profile (False Positive and False Negative Analysis)

Error profile analysis demonstrated variability in false positive (FP) and false negative (FN) rates across models and prompt conditions (Table 5).

Error-direction analysis demonstrated model-specific descriptive patterns. GPT-5 showed the highest false-positive tendency, with false-positive rates of 15.1% under non-prompted and 15.9% under prompted conditions among gold-standard “No” items. Gemini showed consistently low false-positive rates under both conditions (1.6%), but its false-negative rate increased descriptively from 3.7% under non-prompted conditions to 10.2% under prompted conditions among gold-standard “Yes” items. DeepSeek showed a reduction in false-positive responses under prompted conditions, from 10.3% to 6.3%, with no false-negative responses observed under prompted conditions. However, Fisher’s exact tests did not show statistically significant within-model prompt-related changes in false-positive or false-negative rates.

### 3.4. Short-Term Response Stability

**Figure 2:**
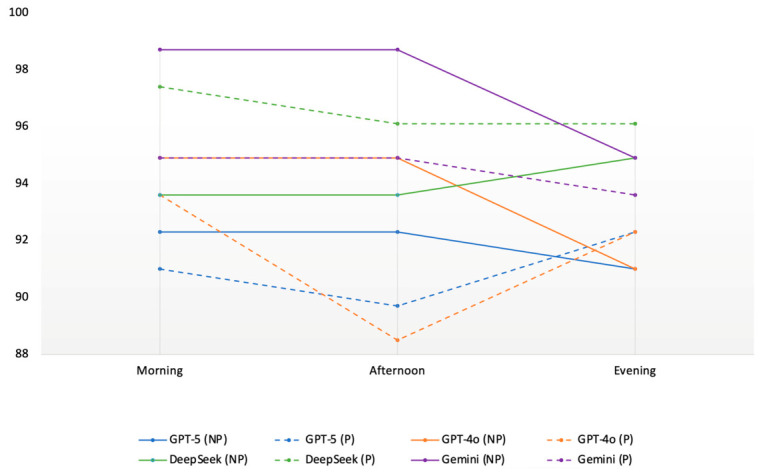
Within-day changes in mean accuracy across models under non-prompted (NP) and prompted (P) conditions.

**Figure 3:**
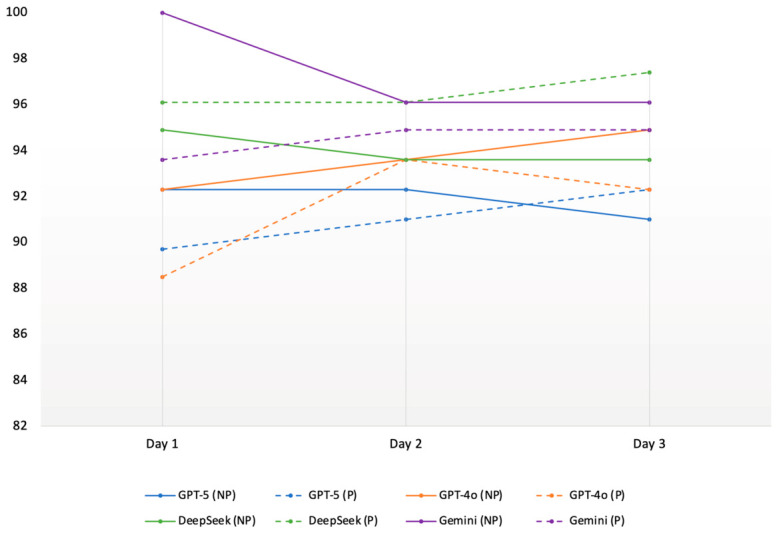
Day-to-day changes in mean guideline-concordance accuracy across chatbot models under non-prompted (NP) and prompted (P) conditions.

In the mixed-effects logistic regression model, neither day nor within-day time point was significantly associated with guideline-concordant response accuracy. Compared with Day 1, the odds of correct responses did not significantly differ on Day 2 (OR = 1.14, *p* = 0.662) or Day 3 (OR = 1.19, *p* = 0.558). Similarly, compared with morning sessions, afternoon and evening sessions were not significantly associated with response accuracy (afternoon: OR = 0.77, *p* = 0.372; evening: OR = 0.71, *p* = 0.239). These findings support short-term response stability across the three-day testing period. Temporal patterns of observed accuracy are additionally illustrated in Figure 2 and Figure 3.

## 4. Discussion

VPT is one of the most diagnostically sensitive procedures in contemporary endodontic practice, with treatment success depending on strict adherence to clinical guidelines and narrow indication thresholds [25]. This study evaluated the ability of widely accessible LLMs to reproduce guideline-defined VPT decision thresholds using a repeated-session, multi-operator design and an item-level clustering-aware mixed-effects analysis. Although all evaluated models demonstrated high overall accuracy (91.02–97.42%), the main finding was not simply that the models performed well on average, but that similar levels of overall performance could mask model-specific differences in guideline-concordance, prompt responsiveness, and decision-level error directionality. After accounting for repeated responses within guideline-derived questions, Gemini showed significantly higher odds of guideline-concordant responses than GPT-5 under non-prompted conditions (OR = 5.73, 95% CI 1.90–17.30), whereas DeepSeek showed significantly higher odds than GPT-5 (Bonferroni-adjusted *p* = 0.018) and GPT-4o (Bonferroni-adjusted *p* = 0.032) under prompted conditions. Accordingly, the null hypothesis was rejected; both model architecture and interaction conditions can lead to clinically meaningful differences in decision support within this protocol-sensitive domain.

In the present study, the ESE position statement was adopted as the reference standard for deep caries management and VPT; recent systematic evaluations have demonstrated its superior methodological quality and clinical applicability among global recommendations [26]. A distinguishing methodological feature of this study is the use of guideline-concordant binary decision statements rather than open-ended clinical scenarios to evaluate model performance. This design was intentionally selected to approximate real-world decision thresholds encountered in evidence-based treatment planning. Previous studies have shown that, when responding to open-ended clinical questions, LLMs may deviate from the target topic or generate speculative information, commonly referred to as hallucinations [23,27,28]. By constraining responses to predefined options derived directly from guideline criteria, the present approach minimized interpretative variability and enabled direct assessment of model adherence to explicit clinical rules. Moreover, this structured framework facilitated transparent classification of false-positive and false-negative responses, allowing a more clinically meaningful evaluation of error directionality and decision-level agreement with established treatment standards.

The evaluated models demonstrated high overall accuracy, with performance ranging between 91.02% and 97.42%. In general, these findings align with a growing body of endodontic literature indicating moderate-to-high LLM performance in guideline-anchored or structured decision tasks, with clinically relevant variability in reliability and reproducibility [29,30,31].

For instance, in a guideline-based assessment focusing on pulp therapy for immature permanent teeth, Sezer & Aydoğdu [29] reported significant performance differences across systems; ChatGPT-4o and DeepSeek outperformed Gemini in both accuracy and completeness, with notable between-model variations observed for response time and readability. Similarly, a case-scenario study by Karaca et al. [30] evaluating diagnostic appropriateness across 50 simulated endodontic cases found substantial performance variation across six LLMs. Accuracy varied significantly by model and decreased in more complex scenarios, reinforcing the notion that LLM outputs are not uniformly reliable across varying clinical contexts and difficulty levels.

The short-term temporal consistency observed in our findings is further supported by Shirani & Emami [31], who compared five LLMs in treatment planning for the restoration of endodontically treated teeth over three consecutive weeks. While Gemini consistently produced the most accurate responses in that specific restorative context, none of the models achieved perfect repeatability, highlighting the inherent volatility of AI outputs.

Several recent studies have evaluated LLM performance in endodontics using different assessment formats. Büker et al. [12] evaluated chatbot accuracy and consistency in endodontic clinical decision support, whereas Çekiç and Tavşan [13] assessed LLM performance using national endodontic specialty examination questions, and Sezer and Aydoğdu [29] evaluated LLM responses in pulp therapy for immature permanent teeth using broader response-quality metrics. In contrast, the present study focused specifically on guideline-defined VPT decision thresholds and combined repeated multi-operator querying, professional-role prompting, short-term stability assessment, false-positive/false-negative error-directionality profiling, and mixed-effects modelling with item-level clustering. Therefore, the present work should be interpreted as complementary to previous endodontic LLM evaluations rather than as another accuracy-only benchmark.

The directionality of errors provides clinically relevant information beyond average accuracy. In this study, false-positive responses represent inappropriate endorsement of guideline-defined statements when the gold-standard answer was “No,” whereas false-negative responses represent rejection of guideline-defined statements when the gold-standard answer was “Yes.” GPT-5 showed a relatively higher false-positive tendency, suggesting a more permissive response pattern. Gemini showed consistently low false-positive rates but a descriptive increase in false-negative responses under prompted conditions, suggesting a more conservative response tendency. DeepSeek showed the most favorable descriptive error profile under prompted conditions, with reduced false-positive responses and no false-negative responses. Although these within-model changes were not statistically significant in Fisher’s exact tests, they highlight that average accuracy may obscure clinically meaningful differences in decision-level risk profiles.

Translating these descriptive error patterns into concrete VPT scenarios further illustrates their clinical relevance. A false-positive response would occur, for example, when a model endorses the statement “vital pulp therapy is indicated for a permanent tooth with a periapical lesion,” a position not supported by the guideline because periapical pathology indicates pulpal necrosis and contraindicates pulp preservation; such an error would extend a conservative treatment approach to a tooth in which root canal treatment was the guideline-concordant choice [22]. Conversely, a false-negative response would occur when a model rejects the statement “in the presence of short-lasting provoked pain and a radiographically normal periapex, vital pulp therapy may be performed,” a guideline-supported indication consistent with reversible pulpitis; such an error could redirect the clinician away from a guideline-concordant pulp preservation option toward root canal treatment, potentially resulting in unnecessary treatment escalation. Across such examples, similar average accuracy may conceal materially different decision-level risk profiles, reflecting distinct patterns of deviation from guideline-concordant decision support. The descriptive patterns observed in this study—particularly the higher false-positive tendency of GPT-5 and the prompted-condition rise in false-negative responses for Gemini—therefore reflect clinically asymmetric risk profiles even where mean accuracy is comparable. Although these scenario-level translations remain illustrative rather than confirmatory and should not be over-interpreted given the non-significant within-model Fisher’s exact tests, they underscore that decision-level error directionality captures the type of clinically meaningful asymmetry that average accuracy may obscure.

An important strength of the present study is the evaluation of short-term response stability across multiple days and repeated query sessions. Short-term stability represents a critical but often underexamined dimension of LLM reliability in clinical decision-support contexts. Because identical clinical queries may be submitted at different time points during routine practice, variability in model responses over time may influence clinician confidence and affect treatment selection. Evaluating within-day and across-day consistency therefore provides insight into whether model outputs can be considered reproducible under repeated exposure to identical decision scenarios. In protocol-sensitive domains such as vital pulp therapy, where treatment recommendations depend on strict adherence to guideline-defined thresholds, even modest fluctuations may have clinically meaningful implications.

In the present study, mixed-effects modelling showed no significant association between response accuracy and either day or within-day time point, supporting relative short-term response stability at the aggregate accuracy level across the three-day testing period. However, this does not exclude item-specific variability or longer-term instability related to future model updates, interface changes, or system-level modifications. Therefore, short-term stability observed under controlled testing conditions should not be interpreted as permanent reproducibility, and LLMs should be regarded as dynamic systems whose outputs may vary over time, further supporting their role as decision-support tools rather than autonomous decision-makers.

Prompt engineering has been proposed as a strategy to refine LLM outputs by providing structured contextual guidance, with the aim of improving accuracy and clinical relevance in health care applications [32]. Our findings indicate that prompt usage has a meaningful but model-dependent impact on guideline-adherence performance.

Consistent with this, a recent guideline-based evaluation in dentistry reported that the effects of system pre-prompts on LLM performance were not uniform or unidirectional [16]. While the application of a pre-prompt improved accuracy in several models, others showed limited improvement or no benefit, and response variability persisted. These findings suggest that prompt engineering may enhance performance under certain conditions, but does not function as a universal optimization strategy.

In line with this, we observed heterogeneous descriptive prompt effects across models, affecting both overall accuracy and the directionality of errors. Prompt usage was associated with modest descriptive reductions in accuracy for GPT-5 and GPT-4o, accompanied by either stable or redistributed false-positive and false-negative rates. In contrast, DeepSeek descriptively improved under prompted conditions, with no false-negative responses observed in the prompted set. Gemini, by comparison, showed a descriptive reduction in accuracy and an increase in false-negative responses under prompted conditions, suggesting a shift toward more conservative decision thresholds. However, the model × prompt interaction did not reach the conventional threshold for statistical significance after accounting for question-level clustering, with wide confidence intervals around individual interaction terms indicating limited precision (Table 4); and Fisher’s exact tests likewise did not demonstrate significant within-model prompt-related changes in false-positive or false-negative rates. These findings should therefore be interpreted as descriptive rather than confirmatory.

Taken together, these results suggest that prompt effects should not be assumed to be uniform, robust, or universally beneficial across LLM architectures. Professional-role prompting may alter response tendencies in clinically relevant ways, but its effects require model-specific validation before being incorporated into routine clinical interpretation.

Notably, GPT-5 did not demonstrate superior overall accuracy compared with the other evaluated models, despite representing the most recently released architecture among those tested. Under non-prompted conditions, GPT-5 exhibited the lowest mean accuracy across all models, and showed a modest further reduction under prompted conditions. This finding contrasts with the general assumption that newer model generations uniformly outperform their predecessors in clinical decision tasks, and suggests that architectural advancement alone does not guarantee improved guideline adherence in protocol-sensitive domains.

A methodological strength of this study is its adherence to the TRIPOD-LLM reporting framework, which provides structured guidance for the transparent reporting of studies evaluating LLM performance in clinical contexts. Compliance with TRIPOD-LLM facilitated the systematic documentation of model selection criteria, experimental conditions, outcome definitions, and analytical procedures, thereby supporting reproducibility and enabling meaningful cross-study comparisons. As the adoption of such reporting standards remains inconsistent in the emerging literature on LLMs in dentistry, the present study may serve as a methodological reference for future investigations in this domain.

A notable methodological feature of the present study is the multi-operator design, in which two independent operators administered identical questions across separate sessions. The majority of existing studies evaluating LLM performance in endodontics rely on single-operator data collection, which may confound operator-specific querying behavior with model-level variability [33]. By incorporating a second operator, the present study enabled clearer attribution of response variability to the models themselves rather than to individual interaction patterns, thereby strengthening the internal validity of the findings.

The findings of this study have important implications for the integration of AI into endodontic practice. Although patient-level outcomes were not assessed, the observed variability in guideline adherence and error directionality highlights a clinically relevant risk that may meaningfully influence treatment selection in daily practice. The high accuracy and relative consistency observed in certain models suggest that LLMs could serve as valuable tools for rapid guideline consultation and educational reinforcement. However, accuracy alone does not equate to clinical safety, and the observed variability in error directionality underscores that LLM-generated recommendations should never be interpreted in isolation. Clinicians must remain aware that some models may favor overtreatment, whereas others may adopt excessively conservative decision thresholds; professional role prompting can subtly shift these model decision boundaries.

Beyond the specific clinical context of VPT, these findings highlight that LLM performance in protocol-driven clinical decision contexts cannot be adequately characterized by overall accuracy alone. Instead, short-term response stability, prompt responsiveness, and error directionality represent critical dimensions influencing the clinical reliability of AI-assisted decision support. This study provides a structured evaluation framework that may be applicable to other guideline-sensitive clinical domains, where consistent adherence to evidence-based decision thresholds is essential. Such multidimensional assessment approaches may contribute to safer and more informed integration of LLMs into clinical workflows.

Several limitations of this study should be acknowledged. First, although the repeated-session design generated a large number of responses, the underlying clinical content was based on 26 guideline-derived items; to reduce the risk of overinterpreting repeated responses as independent observations, a mixed-effects logistic regression model with question ID as a random intercept was incorporated to account for within-item response clustering. In addition, the design was not formally powered for the detection of modest model × prompt interaction effects under question-level clustering; the wide confidence intervals around individual interaction terms (e.g., GPT-4o × prompted OR = 0.73, 95% CI 0.22–2.44; Table 4) indicate limited precision at this level, and the non-significant interaction trend should therefore be interpreted with caution rather than as evidence of equivalence. This non-significant trend (χ^2^ = 6.79, *p* = 0.079) most likely reflects limited statistical power rather than true equivalence between prompt conditions across models. Second, the professional-role prompt used in this study was intentionally simple and did not provide guideline content; therefore, the findings should be interpreted as the effect of role assignment rather than full guideline-informed prompt engineering. Third, false-positive and false-negative analyses were conducted at the guideline-statement level rather than at the patient-outcome level, and therefore represent decision-level risk patterns rather than direct clinical outcomes. Fourth, the use of binary yes/no items was a deliberate methodological choice that allowed direct mapping to explicit guideline-defined decision thresholds and transparent classification of false-positive and false-negative errors; however, this design intentionally simplifies the inherently nuanced and patient-specific nature of real-world VPT decision-making, in which diagnostic interpretation, hemostasis assessment, restorative planning, and patient-level factors interact in ways that cannot be reduced to discrete yes/no judgements. Findings should therefore be interpreted as reflecting LLM performance against explicit guideline rules rather than against complex composite clinical cases. Fifth, the scope of this investigation was limited to VPT and may not be directly generalizable to other complex endodontic procedures, such as apical microsurgery, traumatic injury management, or complex retreatment cases requiring advanced spatial and radiographic interpretation. Sixth, the evaluation was anchored to a single internationally recognized clinical guideline [22]; although this guideline was selected on the basis of demonstrated methodological quality and clinical applicability [26], reliance on a single reference document means that the observed performance reflects concordance with one specific set of evidence-based recommendations rather than the broader space of expert opinion or alternative national, regional, or institutional guidelines. Seventh, all queries were conducted in English; although the evaluated LLMs are multilingual, performance may vary across languages. Eighth, the short-term stability assessment was operationalized as nine query sessions distributed across three consecutive days; this window enables characterization of immediate within-day and across-day variability under a fixed system state but does not extend to longer time horizons. Ninth, all evaluations were conducted through publicly available web interfaces, which more closely reflect routine clinical and educational use than API-based access but introduce uncontrolled exposure to vendor-side backend updates, content filters, and dynamic system instructions that are neither documented nor versioned at the user level. Two identical queries submitted at different timepoints may therefore reach materially different underlying model configurations without external indication, which constrains experimental control and the long-term reproducibility of any LLM evaluation conducted under these conditions. Finally, although short-term response stability was supported within the testing period, the rapid evolution of LLM models means that performance characteristics may change with future updates.

Taken together, the present findings support several practical takeaways for clinicians and for future LLM evaluation in dentistry. For clinicians consulting LLMs in guideline-based decision contexts, the results suggest that (i) overall accuracy benchmarks should not be relied upon as a sole indicator of clinical safety, since models with comparable mean accuracy may carry meaningfully different error directionality profiles; (ii) prompt strategies, including professional-role prompting, should be applied with model-specific awareness, as such prompting altered the direction rather than the magnitude of errors across several of the evaluated models; and (iii) LLM-generated recommendations in protocol-sensitive domains should be cross-checked against the relevant source guideline rather than accepted in isolation, particularly in borderline VPT scenarios involving pulpal status, hemostasis assessment, or material selection.

Future research should address the limitations identified in the present study by incorporating open-ended and scenario-based question formats that more closely reflect real-world clinical complexity. Evaluating LLM performance across multiple languages would enhance the generalizability of findings to non-English-speaking clinical settings. Future studies should also compare routine web-interface-based use with more controlled API-based access, where model versions and generation parameters may be more transparently documented. Longer-term longitudinal evaluations across scheduled and unscheduled model updates would further clarify how stable LLM-generated clinical guidance remains over time. The development of domain-specific evaluation frameworks tailored to guideline-sensitive dental procedures, as well as prospective studies examining the downstream impact of LLM-assisted decision-making on actual treatment outcomes, represent important directions for future investigation.

## 5. Conclusions

Within the limitations of this study, widely accessible LLMs demonstrated high guideline-concordance accuracy in VPT-related decision thresholds. After accounting for repeated responses within guideline-derived questions, model-specific differences remained evident: Gemini performed more favorably under non-prompted conditions (OR vs. GPT-5 = 5.73, 95% CI 1.90–17.30), whereas DeepSeek showed superior performance under professional-role-prompted conditions (Bonferroni-adjusted comparisons against GPT-5 and GPT-4o). The model × prompt interaction showed only a trend toward significance, indicating that prompt responsiveness should be interpreted cautiously. Day and within-day time point were not significantly associated with response accuracy, supporting short-term response stability across the three-day testing period. However, error-direction analysis showed that similar average accuracy may conceal different decision-level risk profiles. These findings support the use of LLMs as adjunctive tools for rapid guideline consultation, but not as autonomous clinical decision-makers. Furthermore, as all evaluations were conducted through publicly available web interfaces, the findings represent a snapshot of model performance at the time of testing; performance characteristics may change as models are updated, and results should be interpreted accordingly. Future evaluations of LLMs in protocol-sensitive clinical domains should extend beyond average accuracy and include item-level variability, prompt responsiveness, short-term stability, and false-positive/false-negative error directionality.

## Figures and Tables

**Table 1:** Summary of inter-observer agreement between Observer 1 and Observer 2 across models and prompt conditions.

Condition	Model	Kappa Range	Agreement Level
Non-prompted	GPT-5	0.922–1.000	Almost perfect
	GPT-4o	0.845–1.000	Almost perfect
	DeepSeek	0.847–1.000	Almost perfect
	Gemini	0.764–1.000	Substantial–Almost perfect
Prompted	GPT-5	0.922–1.000	Almost perfect
	GPT-4o	0.692–1.000	Substantial–Almost perfect
	DeepSeek	0.923–1.000	Almost perfect
	Gemini	0.845–1.000	Almost perfect

**Table 2:** Accuracy performance under non-prompted (NP) conditions.

		Confidence Interval		
Models	Mean Accuracy (%)	Lower Limit	Upper Limit	SD
GPT-5	91.87	90.9	92.85	1.26
GPT-4o	93.56	91.50	95.63	2.68
DeepSeek	94.00	91.40	96.59	3.37
Gemini	97.42	94.85	99.98	3.33

**Table 3:** Accuracy performance under prompted (P) conditions.

		Confidence Interval		
Models	Mean Accuracy (%)	Lower Limit	Upper Limit	SD
GPT-5	91.02	88.93	93.11	2.71
GPT-4o	91.86	89.56	94.17	3.00
DeepSeek	96.53	95.53	97.53	1.30
Gemini	94.41	92.87	95.95	2.00

**Table 4:** Mixed-effects logistic regression analysis for guideline-concordant response accuracy.

Predictor	OR	95% CI	*p* Value
GPT-4o vs. GPT-5	1.50	0.62–3.64	0.370
DeepSeek vs. GPT-5	1.68	0.68–4.12	0.258
Gemini vs. GPT-5	5.73	1.90–17.30	0.002
Prompted vs. non-prompted	0.84	0.36–1.92	0.672
Day 2 vs. Day 1	1.14	0.64–2.01	0.662
Day 3 vs. Day 1	1.19	0.67–2.11	0.558
Afternoon vs. morning	0.77	0.43–1.38	0.372
Evening vs. morning	0.71	0.39–1.26	0.239
GPT-4o × prompted	0.73	0.22–2.44	0.607
DeepSeek × prompted	2.78	0.72–10.70	0.138
Gemini × prompted	0.40	0.10–1.63	0.198

**Table 5:** Distribution of false positive (FP) and false negative (FN) responses across days and within-day time points under non-prompted and prompted conditions.

Model	Prompt Condition	False Positive n/N (%)	False Negative n/N (%)	Total Error (%)
GPT-5	Non-prompted	19/126 (15.1)	0/108 (0.0)	8.1
GPT-5	Prompted	20/126 (15.9)	1/108 (0.9)	9.0
GPT-4o	Non-prompted	14/126 (11.1)	1/108 (0.9)	6.4
GPT-4o	Prompted	15/126 (11.9)	5/108 (4.6)	8.5
DeepSeek	Non-prompted	13/126 (10.3)	1/108 (0.9)	6.0
DeepSeek	Prompted	8/126 (6.3)	0/108 (0.0)	3.4
Gemini	Non-prompted	2/126 (1.6)	4/108 (3.7)	2.6
Gemini	Prompted	2/126 (1.6)	11/108 (10.2)	5.6

## Data Availability

The data supporting the findings of this study are available from the corresponding author upon reasonable request.

## Supplementary Materials

The following supporting information can be downloaded at: https://www.mdpi.com/article/10.3390/healthcare14121605/s1, Table S1: Guideline-derived vital pulp therapy decision statements and corresponding gold-standard answers used for model evaluation.

## Abbreviations

The following abbreviations are used in this manuscript:

AI	Artificial Intelligence
LLM	Large Language Model
VPT	Vital Pulp Therapy
ESE	European Society of Endodontology
NP	Non-Prompted Condition
P	Prompted Condition
FP	False Positive
FN	False Negative
SD	Standard Deviation
ICC	Intraclass Correlation Coefficient
SPSS	Statistical Package for the Social Sciences
TRIPOD-LLM	Transparent Reporting of a Multivariable Prediction Model for Individual Prognosis or Diagnosis Using Large Language Models
