# Supplementary material for: How Reliably Do Large Language Models Reproduce Vital Pulp Therapy Guidelines? A Mixed-Effects Evaluation of Guideline-Concordance and Error Directionality
Source: Healthcare (Basel). 2026 Jun 7;14(12):1605. doi: 10.3390/healthcare14121605 (PMC13299513; doi:10.3390/healthcare14121605)
Supplement: Supplementary file 1 [file healthcare-14-01605-s001.zip › healthcare-4320630-supplementary.pdf]

Supplementary Table S1. Guideline-derived vital pulp therapy decision statements used for model evaluation

Question No	Statement	Correct answer
Q1	In selective caries removal, some soft dentin may be left on the pulpal wall.	Yes
Q2	Performing pulp vitality tests is not necessary when planning vital pulp therapy in asymptomatic teeth.	No
Q3	The use of rubber dam in vital pulp therapy influences the success of the treatment.	Yes
Q4	During vital pulp therapy, hydraulic calcium silicate material should be placed directly over the exposed pulp.	Yes
Q5	MTA and bioceramic materials provide a higher success rate in vital pulp therapy compared with calcium hydroxide.	Yes
Q6	Calcium hydroxide continues to be recommended as the gold standard material for vital pulp therapy.	No
Q7	Adhesive resin-based materials (bonding, composite) should not be applied directly to the pulp.	Yes
Q8	In the presence of short-lasting provoked pain and a radiographically normal periapex, vital pulp therapy may be performed.	Yes
Q9	Vital pulp therapy is indicated for a permanent tooth with a periapical lesion.	No
Q10	Vital pulp therapy is not performed in the presence of uncontrolled bleeding or necrotic tissue in the pulp chamber.	Yes
Q11	Sodium hypochlorite (0.5–5%) can be used for both haemostasis and disinfection in vital pulp therapy.	Yes
Q12	Chlorhexidine is not used in vital pulp therapy.	No
Q13	Physiological saline is considered sufficient as the sole agent for disinfection and hemostasis in vital pulp therapy.	No

Question No	Statement	Correct answer
Q14	If pulpal bleeding cannot be controlled within 5 minutes, vital pulp therapy cannot be performed.	No
Q15	Vital pulp therapy does not require completion of the final restoration in the same appointment.	No
Q16	Vital pulp therapy is indicated only in immature permanent teeth.	No
Q17	Periapical radiographic follow-up limited to the first 6 months is sufficient after vital pulp therapy.	No
Q18	Vital pulp therapy success can be confirmed without radiographic follow-up if the tooth is asymptomatic.	No
Q19	The duration and color of pulpal bleeding are important parameters for assessing pulp vitality during vital pulp therapy.	Yes
Q20	Vital pulp therapy performed after traumatic pulp exposure has a higher success rate than after carious pulp exposure.	Yes
Q21	Vital pulp therapy should always be abandoned in cases of irreversible pulpitis.	No
Q22	The size of the pulp exposure is the primary determinant of whether vital pulp therapy can be performed.	No
Q23	CBCT is required as a routine diagnostic tool before performing vital pulp therapy.	No
Q24	Vital pulp therapy can support continued root development (apexogenesis) in immature teeth.	Yes
Q25	The outcome of vital pulp therapy is highly dependent on achieving a durable coronal seal.	Yes
Q26	In traumatic pulp exposures, total pulpectomy is the primary treatment option.	No
